# Pharmacists' Perceptions of the Barriers and Facilitators to the Implementation of Clinical Pharmacy Key Performance Indicators

**DOI:** 10.1371/journal.pone.0152903

**Published:** 2016-04-04

**Authors:** Laura V Minard, Heidi Deal, Megan E Harrison, Kent Toombs, Heather Neville, Andrea Meade

**Affiliations:** 1 Department of Pharmacy, Nova Scotia Health Authority, QEII Health Sciences Centre, Halifax, Nova Scotia, Canada; 2 College of Pharmacy, Dalhousie University, Halifax, Nova Scotia, Canada; Mayo Clinic, UNITED STATES

## Abstract

**Background:**

In hospitals around the world, there has been no consensus regarding which clinical activities a pharmacist should focus on until recently. In 2011, a Canadian clinical pharmacy key performance indicator (cpKPI) collaborative was formed. The goal of the collaborative was to advance pharmacy practice in order to improve patient outcomes and enhance the quality of care provided to patients by hospital pharmacists. Following a literature review, which indicated that pharmacists can improve patient outcomes by carrying out specific activities, and an evidence-informed consensus process, a final set of eight cpKPIs were established. Canadian hospitals leading the cpKPI initiative are currently in the early stages of implementing these indicators.

**Objective:**

To explore pharmacists' perceptions of the barriers and facilitators to the implementation of cpKPIs.

**Methods:**

Clinical pharmacists employed by the Nova Scotia Health Authority were invited to participate in focus groups. Focus group discussions were audio-recorded and transcribed, and data was analyzed using thematic analysis.

**Findings:**

Three focus groups, including 26 pharmacists, were conducted in February 2015. Three major themes were identified. *Resisting the change* was comprised of documentation challenges, increased workload, practice environment constraints, and competing priorities. *Embracing cpKPIs* was composed of seeing the benefit, demonstrating value, and existing supports. *Navigating the unknown* was made up of quality versus quantity battle, and insights into the future.

**Conclusions:**

Although pharmacists were challenged by documentation and other changes associated with the implementation of cpKPIs, they demonstrated significant support for cpKPIs and were able to see benefits of the implementation. Pharmacists came up with suggestions for overcoming resistance associated with the implementation of cpKPIs and provided insights into the future of pharmacy practice. The identification of barriers and facilitators to cpKPI implementation will be used to inform the implementation process on a local and national level.

## Introduction

In Canada and other countries around the world, the role of the pharmacist has been rapidly evolving from a traditional role in drug distribution to expanded clinical roles such as making medication-related recommendations to other members of the health care team, identifying drug therapy problems, assessing patients and prescribing medications, and administering medications such as vaccines [1]. Randomized controlled trials (RCTs) have demonstrated that inpatient, health care team-based pharmacists providing proactive patient care services, significantly reduce the number of hospital visits, the rate of hospital readmissions and costs of hospital care [2,3]. In New Zealand, Ng and Harrison sought to identify a set of measurable key performance indicators (KPIs) to demonstrate the contribution of hospital clinical pharmacy to patient care [4]. Although the study highlighted a number of potential KPIs for hospital pharmacy practice, questions remained about the measurability, evidence-base, and applicability of the select KPIs. Therefore, until recently, there has been no consensus, nationally or internationally, regarding which clinical activities pharmacists should focus on in the hospital system.

In 2011, a national clinical pharmacy key performance indicator (cpKPI) collaborative was formed in Canada in cooperation with the Canadian Society of Hospital Pharmacists with the goal of advancing pharmacy practice to improve patient outcomes. Twenty-six clinical pharmacists and hospital pharmacy leaders developed a final core set of eight cpKPIs through a modified Delphi survey consensus process as described in detail by Fernandes et al [5]. To guide this process, a five-point cpKPI definition was established. Each cpKPI had to 1) reflect a desired quality practice, 2) link to direct patient care, 3) have evidence supporting an impact on meaningful patient outcomes, 4) be pharmacy or pharmacist sensitive, and 5) be feasible to measure. In 2013, via a systematic national evidence-informed consensus process, a core set of eight cpKPIs were established: 1) performing admission medication reconciliation (including best possible medication history), 2) participating in interprofessional patient care rounds, 3) completing pharmaceutical care plans, 4) resolving drug therapy problems, 5) providing in-person disease and medication education to patients, 6) providing discharge patient medication education, 7) performing discharge medication reconciliation, and 8) providing bundled, proactive direct patient care activities (bundle of interventions required for continuous pharmaceutical care from admission to discharge) [5]. The cpKPIs were informed by a literature review that demonstrated that by carrying out these clinical activities pharmacists can improve patient outcomes [2, 3, 6–8]. For example, when pharmacists provide bundled, proactive direct patient care activities (cpKPI #8), hospital readmissions are reduced [2, 3]. This specific cpKPI, also known as the "bundle", includes five interlinked processes of care: pharmaceutical care plan and/or resolution of drug therapy problems, admission medication reconciliation, active participation on interprofessional rounds, patient education (during hospital stay and/or at discharge), and discharge medication reconciliation. The inclusion of this cpKPI recognizes that the RCT evidence that demonstrated a positive impact on patient care involved pharmacist delivery of a "bundle" of interrelated interventions rather than the isolated elements [2, 3, 5].

Canadian hospitals leading the cpKPI initiative are in the early stages of implementing these indicators; implementation of cpKPIs at the Nova Scotia Health Authority, Central Zone (NSHA; formerly Capital District Health Authority), began in October 2014 and will continue over a three-year period. The implementation plan for year 1 involved the implementation of three cpKPIs (performing admission medication reconciliation, performing discharge medication reconciliation, and resolving drug therapy problems) in one or two clinical pharmacy areas at a time. Implementation was led by a cpKPI staff pharmacist and involved: 1) adaptation of a current workload documentation tool (Emerald) [9] used by pharmacists providing direct patient care to record cpKPI activities, 2) dedicated discussions about the three cpKPIs for year 1, and 3) interventions to optimize pharmacist involvement in these activities. At the time of this research, implementation had begun in approximately half of all clinical areas.

Emerald was chosen for documentation as it was the electronic system already in use at NSHA for workload documentation by pharmacists. Since the tool could be adapted to include the specific cpKPI activities, pharmacists would continue to have a single system in which workload could be recorded. Documentation in Emerald includes generation of a patient list each day by selecting all patients that the pharmacist has encountered. For each patient encounter, pharmacists record the amount of time they spent performing specific activities. These activities primarily include the cpKPI activities (for example, performing admission medication reconciliation or patient medication education), but can also include non-cpKPI activities such as providing immunizations. Pharmacists also record activities such as attending departmental meetings or educational events. Pharmacists are expected to use Emerald each day to document workload, and their goal is to provide these evidence-based cpKPI activities to as many patients as possible. Data from Emerald is summarized and reported by the cpKPI staff pharmacist at clinical team meetings. This data is then discussed and used to design interventions that aim to help front-line pharmacists achieve their goal.

Clinical pharmacists' thoughts and opinions about the implementation of cpKPIs have not yet been explored in the literature. We therefore conducted formal qualitative research using focus groups to explore pharmacists' perceptions of the barriers and facilitators to the implementation of cpKPIs.

## Methods

### Study Design

This qualitative study used focus groups to explore pharmacists' perceptions of the barriers and facilitators to the implementation of cpKPIs at NSHA. Focus groups have been used to examine a variety of pharmacy practice issues [10–12]. A focus group design to study pharmacists' perceptions was particularly appropriate since "one premise related to the use of focus groups is that attitudes and perceptions are not developed in isolation, but through interaction with other people" [13]. Advantages of focus groups include a safe environment for participants to discuss perceptions, ideas, and opinions; participants are able to contribute as little or as much as they wish; participants may be more willing to discuss particular topics in a group setting where members of the group may provide support for one another; and participants are able to compare, contrast, and revise their views based on those of others [12, 14, 15]. In addition, focus groups allow for interactions that occur between participants to be studied, and group discussions provide direct information about similarities and differences in participants' opinions and experiences [16]. Ethics approval was granted by the Capital District Health Authority Research Ethics Board (CDHA-RS/2015-235).

### Participants

Focus groups were formed from the pre-existing group of NSHA-employed clinical pharmacists using purposive sampling. Clinical pharmacists employed by NSHA were invited via email to participate in a single focus group at a location within NSHA external to the pharmacy department. Verbal announcements about the research study were made at cpKPI Leadership Team meetings. The study was also advertised at all NSHA sites that employ at least one clinical pharmacist: Queen Elizabeth II Health Sciences Centre (Halifax Infirmary and Victoria General sites) (n = 46), Dartmouth General Hospital (n = 9) and Hants Community Hospital (n = 1).

At the time this research was conducted, there were 56 clinical pharmacists employed by NSHA; however, clinical coordinators who supervise clinical pharmacists (n = 5) were excluded from this study to avoid differences in authority and status within each group and to improve the likelihood that participants would freely contribute to the discussion [17].

### Focus Group Structure and Moderation

Focus groups were moderated by the principal investigator (LVM) and assistant moderator (MEH). Only the moderator, assistant moderator and participants were present. Focus groups were between 70 and 97 minutes in duration. Prior to the focus group sessions, participants were provided with an information package that outlined background information on cpKPIs and the implementation plan at NSHA. All participants provided written informed consent and signed a pledge of confidentiality prior to participating in this study as approved by the Capital District Health Authority Research Ethics Board. The principal investigator and assistant moderator also signed a pledge of confidentiality. Given the confidential nature of this research, focus group transcripts were not returned to participants for review. Rather, participant verification occurred at the end of each focus group by asking the question "What do you think the most important elements of this discussion have been?" [18].

### Data Collection and Analysis

Short and broadly focused questions were selected from a semi-structured topic guide (S1 Appendix) [19]. Examples of specific questions and prompts that were used are presented in S1 Appendix; however, the topic guide served only as an outline with the aim of generating discussion that would help to address the research question [16]. To further encourage discussion among focus group participants, one rating question using a predetermined scale was included (S1 Appendix) [20]. Participants independently rated their level of agreement with the statement and then discussed tabulated group results.

Focus group discussions were audio-recorded and transcribed by the principal investigator. Transcript-based analysis, the most rigorous and time-intensive method of focus group data analysis [21], was conducted alongside field notes constructed by the moderator and assistant moderator [16]. Thematic analysis was used to examine pharmacists' perceptions of facilitators and barriers to the cpKPI implementation process. Thematic analysis of focus group transcripts was an iterative process that involved open coding of text, the grouping of codes into categories, and the generation of themes that represent the content of each of the categories [22]. Specifically, codes were created by applying a label to data that was discerned as a pattern among otherwise random information. Related codes were grouped together resulting in categories, and the linking of related codes and categories led to the development of themes. During each iterative cycle, transcripts, codes, categories and themes were compared in all directions; transcripts were re-coded and re-categorized as themes emerged from the data [23]. Coding was conducted by the principal investigator and reviewed with one co-investigator who was not employed by NSHA (HD). This involved discussion regarding the codes to uncover any differences in data interpretations and further explore any emerging themes of the study. This is referred to as peer debriefing. NVivo 10 software was used to facilitate transcript-based analysis and thematic analysis [24].

### Rigour and Trustworthiness

Several measures were taken to maintain trustworthiness of the research findings. A reflexive approach was used by the principal investigator to acknowledge and make transparent personal thoughts, concerns, and values that could influence the interpretation of the data [25]. The principal investigator wrote in a reflexive journal after each focus group, during transcription, and throughout thematic analysis. This journal was used to document thoughts about focus groups and participants, unexpected participant comments, focus group discussion flow, missed opportunities for the moderator to probe more deeply into participant comments, and personal biases. The journal was also used to record ideas for new codes and categories and was reviewed periodically to help refocus analysis. For example, review of the journal in the early stages of thematic analysis identified that many participants seemed confused by the word "implementation". The following is an excerpt from the journal of the principal investigator:

I had to bite my tongue several times. Much of the group seemed unfamiliar with what 'implementation' meant. Most group members had not formally begun implementation, but do perform these activities as part of their daily jobs. I did clarify eventually what was meant by 'implementation' as it had come up multiple times. Not sure if I should have done this, and not sure if it helped.

This provided context for participant comments throughout data analysis (see Findings and Discussion). The principal investigator is a researcher as well as a clinical pharmacist (BSc, PhD, BSc(Pharm)); therefore, it was particularly important to use this journal to maintain self-awareness of these two intertwining roles and to consider how this, or any personal biases, may affect interpretation of the data. At the time of this research, the principal investigator was a pharmacy resident and had an established relationship with most participants. Participants were well-informed about research goals and moderator characteristics.

To increase credibility and reliability of the research findings, the principal investigator debriefed with the assistant moderator after each focus group; engaged in continuous reflection and re-examination of the data, codes, categories, and themes throughout multiple cycles of data analysis; and participated in peer debriefing with one co-investigator (who was not employed by NSHA) to challenge and explore interpretations of the data. The research context, selection of participants, data collection, and analysis have been clearly described to facilitate transferability of the findings. Memos and notes detailing the history and development of codes to categories and themes were documented to provide an audit trail. In addition, participant quotations were included to increase trustworthiness by demonstrating links between the original data and categories or themes [26].

## Findings

Three focus groups, including a total of 26 clinical pharmacists, were conducted in February 2015. Twenty-five clinical pharmacists did not participate: 16 pharmacists could not participate due to scheduling conflicts or other work priorities, six pharmacists did not respond, and three pharmacists declined. A broad comparison of each focus group is shown in Table 1. Focus groups contained between six and 12 participants. After transcribing and coding all focus groups, a total of 130 unique codes were identified (S2 Appendix). When codes were compared between groups, there was 67.6% to 83.0% overlap.

**Table 1:** Comparison of focus groups: number of participants, number of new codes identified and total number of codes identified within each group.

	Focus group A	Focus group B	Focus group C	Total
**Number of participants**	8	6	12	26
**Number of new codes**	108	20	2	130
**Total number of codes**	108 (83.1%)	93 (71.5%)	88 (67.7%)	130 (100%)

Using an iterative process, 130 codes, nine categories and three major themes were identified from the focus groups. The three major themes were: *resisting the change*, *embracing cpKPIs*, and *navigating the unknown*.

### Resisting the Change

Pharmacists resisted change associated with the implementation of cpKPIs. Resistance was mainly due to challenges related to documenting clinical activities, as well as to increased workload, practice environment constraints, and competing priorities.

### Documentation Challenges

A significant amount of time in each focus group was spent discussing the challenges of documenting clinical activities using Emerald [9]. Emotions including aggravation, frustration, guilt, and resentment were associated with the process of documentation. For example, one participant stated, "it's just the *details* that are aggravating people" (Participant A2). Another participant stated, "I find some people are recording in different areas and it just gets frustrating when you see other people's stats and you don't know if you're on the same level or if you're doing it the same way" (Participant C1). Pharmacists felt that the requirement for continuous documentation of clinical activities was unnecessary, difficult to maintain, and tiresome: "I made Emerald my work New Year's resolution. I think I was good for two weeks and then unfortunately I fell off the wagon" (Participant C3). Pharmacists also found the process confusing due to unclear definitions of cpKPIs (e.g. drug therapy problems resolved).

Additional barriers to the documentation process included the need to account for the time spent to complete each activity and to attach this time to an individual patient, a large number of patients to record activities for, difficulty tracking daily clinical activities, and forgetting to document clinical activities. One pharmacist stated: "It's hard to keep track…of *all* the different…questions that you're dealing with or different scenarios, trying to document everything…it is hard to kinda track everything that you're doing at such a pace" (Participant A6). Barriers related to the Emerald tool itself included too much clicking and scrolling, and problems selecting a patient list, particularly for pharmacists who work in areas with off-service patients:

We have to manually go in and pick our whole patient load.

(Participant A7)

Which changes every day.

(Participant A5)

Yeah it's kind of the problem with anybody that has patients on multiple floors. You can't just pick a *service*. You have to pick a floor.

(Participant A7)

There were significant concerns regarding the validity of the data collected from Emerald (e.g. inconsistent recording of activities among pharmacists) as well as fear of job loss, for example, if the data collected does not demonstrate pharmacist value.

So what if they get these numbers and the numbers show that…we're not getting the bang for the buck? Then they'll say 'okay well we *don't* need a pharmacist there because they're really *not* making an impact'…because it didn't look like you were doing anything for them just based on the way you were tracking it.

(Participant A3)

### Increased Workload

Pharmacists identified increased workload as a barrier to the implementation process; this was in large part due to time: “I find my biggest barrier is just finding the time to do the Emerald…” (Participant B6). Time-related barriers included the time required to document clinical activities in Emerald, and the time required to document good quality data that accurately reflects daily activities and can be used for case costing purposes.

Pharmacists also felt overwhelmed by the increased workload and stated that it is difficult to do all eight of the cpKPIs. They also felt frustrated and overloaded with things to do, especially in light of too much other change in the department (e.g. decentralized order entry, the merger of inpatient and outpatient dispensaries at one site).

I think the pharmacists do want to try, but I also think we're getting overloaded with a lot of stuff. It's just new things all the time, and it's just, it's one more thing to add on the caseload.

(Participant A5)

### Practice Environment Constraints

Inconsistent clinical pharmacist coverage as a barrier to the implementation of cpKPIs was highlighted by the comment, "if we're only there one or two days a week, you know, you do what you can do, but obviously there's a lot that's getting missed" (Participant C4). Practice environment constraints such as pharmacist hours worked, a chaotic work environment, not enough money or resources (e.g. computers), and a large number of patients to follow were also identified as barriers to the implementation of cpKPIs. For example, participants in one group echoed agreement with one pharmacist who stated, "I think about [one floor], like if you guys had, instead of 30 patients if you had eight patients, you would get everything done for everybody" (Participant C8). Difficulty finding things (e.g. patient chart, medication administration record) and patient location (e.g. off-service patients more difficult to reach) were also obstacles to the ability of pharmacists to perform cpKPIs.

I'd hate to see the stats on our off-service patients…I mean they're not getting seen by me nearly as often as the people on [my own floor] because of the physical barrier of trying to get there. It's sad, but true.

(Participant B1)

Pharmacists outlined several differences between sites and clinical areas that can impede the ability of pharmacists to implement cpKPIs. For example, admission or discharge medication reconciliation may not be as relevant in certain inpatient settings as others.

If I try to do med rec on every patient, I feel sometimes like it's not the best use of my time 'cause the patients might not necessarily be able to communicate, and I'm almost making more work in the end because then someone might have to re-do my work and then I haven't focussed as much time on my pharmaceutical care plan, for example.

(Participant B2)

Pharmacists also mentioned the difficulty of finding an appropriate time to interact with patients (e.g. patient gone for surgery, patient in pain or sedated), which can differ between clinical areas.

Specific constraints related to medication reconciliation included problems using the hospital's electronic discharge medication reconciliation tool (e.g. inability of pharmacist to suggest changes online), the timing of discharge medication reconciliation (e.g. too soon or too late depending on the clinical area), no best possible medication history completed for admitted patients, and problems created by joint competencies with other healthcare professionals (e.g. neither nursing or pharmacy takes full responsibility for the joint competency of admission medication reconciliation).

### Competing Priorities

Resistance to the implementation of cpKPIs was partly due to the wide variety of competing priorities facing pharmacists.

What about our other expectations that we're supposed to be doing like order entry that's not negotiable, that has to be done? So that kind of gets away from your ability to…

(Participant B5)

Work on KPIs.

(Participant B1)

Yeah, and like research projects…am I supposed to not do that one day and enter my Emerald instead? And then I'd feel bad that I didn't help out with the research project that's ongoing so there's some things that aren't on the KPIs, but they have to be done day by day.

(Participant B5)

Other competing priorities included distribution activities, coverage of the hospital outpatient pharmacy, attendance at rounds, and activities expected by other health care professionals that are not established cpKPIs (e.g. writing orders, completing medication calendars). Pharmacists wondered if their time was best spent implementing cpKPIs when compared with other activities they perceived as valuable such as teaching and precepting learners: "Yeah I find it hard to make that call or decision sometimes. Like should I be teaching more right now or should I be trying to get some of these KPIs done?" (Participant B2).

A substantial amount of discussion focussed on the time spent documenting in Emerald versus spending this time on other activities. "You could argue that less patients are seen…maybe that's one less patient I see that day to record my Emerald…” (Participant C12). Pharmacists also considered whether cpKPIs are already outdated and questioned whether there are other activities they should now be performing.

Yeah the KPIs aren't moving along with what we're doing necessarily…so as new things come up that we think are important, like antimicrobial stewardship or research [or] different things. The KPIs are staying the same.

(Participant B2)

### Embracing Clinical Pharmacy Key Performance Indicators

Although pharmacists resisted change associated with the cpKPIs, pharmacist support for the clinical activities outlined by the cpKPIs was clear. Evidence that pharmacists embraced cpKPIs came from their ability to see the benefit of the cpKPIs, their thoughts that cpKPIs may help to demonstrate pharmacist value, and the identification of supports already in place at the organization that facilitate the implementation of cpKPIs.

### Seeing the Benefit

I like the idea that we are trying to focus on activities that [are] supported in the literature. So I think that that's good…and it's exciting that we're trying to review our practice and make sure that what everyone's doing is, you know, consistent and reliable.

(Participant A2)

Pharmacists identified that one major benefit of cpKPIs was the potential for them to help focus pharmacist clinical activities. This was closely linked to the idea that current pharmacy practice is inconsistent and that the implementation of cpKPIs may lead to a more consistent practice. Pharmacists felt that this would help align expectations of other health care professionals and help identify activities that may be shifted to others. Pharmacists also noted that cpKPIs allow them to re-focus or remind them what needs to be done. Opinions indicating support for cpKPIs were voiced and included cpKPIs are important, cpKPIs make sense, cpKPIs are evidence-based, there is value in the implementation, and cpKPIs may benefit patient care. Some pharmacists also felt excited about cpKPIs and were hopeful that they will benefit practice.

Pharmacists felt that the barriers to implementing cpKPIs could be managed or overcome. Two pharmacists also indicated that their initial negative impressions of the implementation of cpKPIs had become more positive once they had actually begun the formal implementation process.

Pharmacists indicated that there is a culture of support for cpKPIs at NSHA. Specifically, when pharmacists were asked to rate their level of agreement with the statement "Pharmacists at NSHA support the implementation of cpKPIs" (S1 Appendix), five pharmacists selected 'to a great extent', 17 pharmacists selected 'to a moderate extent', four pharmacists selected 'to a slight extent' and no pharmacists selected 'not at all'.

I think most pharmacists would agree that we should *do* these things. It's just like actually the *process* of reporting it, that I think there would be more people's lack of support for that than actually carrying out the KPIs.

(Participant B3)

There was discussion in each group regarding the idea that cpKPIs are what pharmacists already do, and unexpectedly, there was confusion in all focus groups regarding the word "implementation". Pharmacists felt that they are performing the same activities they have always been performing, but they recognized that now they have to document and monitor these activities.

I'm struggling with the implementation, the term implementation of KPIs…because this is my practice, and it's been my practice for ten years…these eight are mostly what I did anyway, so the only thing that's changed in my practice since we've implemented these is that I now have to document that I'm doing it.

(Participant A7)

### Demonstrating Value

I think it’s important as clinical pharmacists that we are constantly being told to prove ourselves, and prove what we do, and prove our worth, that maybe by starting to document what we *actually* do because that's a big part of what's missing…people don't actually know what we do…that it might help.

(Participant A7)

Pharmacists recognized that documentation of clinical activities is important. Some pharmacists agreed that having this data may allow pharmacists to demonstrate value, which could justify staff positions or lead to positive change.

Maybe if they look at the data and see that you're only targetting so many med recs, so many discharges…how can we get more pharmacist time up there? I know that's being very Pollyanna about it, but maybe they'll look at it and realize that we're stripped too thin now. We're not getting nearly the numbers we should be.

(Participant B5)

Pharmacists also indicated that cpKPIs may increase patient awareness of the role of the pharmacist and help to define and resolve confusion over the pharmacist's role among other health care professionals. Pharmacists discussed the benefits of being able to show what they do and that cpKPIs can be seen as a "way to quantify pharmacist value" (Participant C8).

### Existing Supports

Pharmacists were able to identify several supports that were put in place prior to beginning the cpKPI implementation process that facilitated their ability to implement cpKPIs. These included a dedicated staff pharmacist to lead the cpKPI initiative, support of the organization, technical support, improvements to Emerald including descriptions within Emerald to guide pharmacists on how to enter clinical activities, and Emerald training sessions.

Pharmacists identified management-led facilitators of the cpKPI initiative including reminders to enter workload in Emerald and being held accountable to enter workload. Pharmacists also identified facilitators of the cpKPI initiative that were led by the cpKPI staff pharmacist such as discussions within each clinical group to trouble-shoot and problem solve issues as they arose in real time (e.g., to clarify how to enter specific tasks into Emerald), promotion of the initiative to members of the interprofessional patient care team, and follow-up and feedback.

Having someone like [the dedicated cpKPI staff pharmacist] actually parsing through the numbers and trying to make sense of it…[has] really been the thing that has made me submit my data much more on a regular basis than before. It's just nice to know that it's not just data that you're doing because someone tells you you should be doing it. You can hopefully see that someone's gonna analyze it and make some sense of it.

(Participant B4)

A number of initiatives led by front-line clinical pharmacists were also identified as facilitators of the implementation of cpKPIs including the development of tools to assist with the tracking of clinical activities, providing handover to other clinical pharmacists, observing and learning from peers, and estimating a standard amount of time that each task takes to ease the documentation process. Some pharmacists also felt that documentation itself can serve as a reminder of activities that have been done as well as highlight those left to be completed. Communication with other health care professionals to promote pharmacist involvement in patient care activities as defined by the cpKPIs was also identified as a facilitator.

I've talked to staff about what I'm looking for, like the nursing staff, like if you have any med recs that need to be done or problems with their meds, come to me right away. So they do kind of come to me now and say 'We need some help here'. So I have to tell them what I'm looking for to focus on it, I suppose. It seems to have helped.

(Participant A8)

Some pharmacists also recognized that joint competencies with other health care professionals can support pharmacist clinical activities (e.g. pharmacy technician completion of best possible medication histories prior to patient admission). One pharmacist acknowledged that learning about the national cpKPI initiative early facilitated the implementation process.

### Navigating the Unknown

In addition to discussing the facilitators and barriers to the implementation of cpKPIs, pharmacists reflected on issues that are currently a source of uncertainty such as how to address the "quality versus quantity battle" and what cpKPIs will mean for the future of pharmacy practice.

### Quality versus Quantity Battle

"Do you try to do the most for a smaller number of patients…or do you try to just do a limited amount of care for a larger number of patients"? (Participant A2). Given that the number of patients assigned to each pharmacist can be as high as 36 or more in some clinical areas, pharmacists reflected on whether they should provide comprehensive care to a smaller number of patients or superficial care to a larger number of patients. This question of the "quality versus quantity battle" was also complicated by considering how to prioritize patients given that the complexity of each patient and each cpKPI differs.

I might spend two or three hours on one really complex problem, and it might have saved someone's life, and then the other guy I might have spent five minutes and solved six things right away, so that in my mind sometimes is the battle.

(Participant B3)

While pharmacists recognized that not every patient needs every cpKPI, there was genuine desire from pharmacists to provide care to and impact a larger number of patients.

### Insights into the Future

Pharmacists' insights into the future of cpKPIs and pharmacy practice spontaneously arose during each focus group in spite of a lack of questioning from the moderator. In particular, pharmacists discussed the validity of the Emerald data at these early stages of implementation, how this data will be reported, what the data will mean for pharmacists in the future, and whether pharmacist jobs will be affected by this data. In light of the recent merger into one provincial health authority in Nova Scotia, pharmacists wondered whether the cpKPI initiative will soon become a priority throughout the province.

There was also conversation regarding whether pharmacists should have the individual right to choose which clinical activities they focus on or whether activities should be streamlined. Some pharmacists felt that they use clinical judgment to help them prioritize and care for patients whereas others felt that streamlining practice will make practice more consistent and align expectations between pharmacists and other health care professionals. There was some concern that unless updated, the cpKPIs, which were established in 2013, could limit pharmacy practice going forward and prevent pharmacists from practicing within an expanded scope.

If we're trying to expand our *profession* or what we do, like, that's one of the things about designing these KPIs. It's nice to know what we *do* and what we want to focus on, but we don't want to be limited to this.

(Participant C2)

Additional unknowns included what the cpKPI targets will be, whether the quality of each cpKPI will be measured, how cpKPIs will be evaluated, which cpKPIs are "most" evidence-based, and whether the cpKPI initiative will lead to practice change.

I'm hopeful. I have hope it will be beneficial, but I guess I'm trying to figure out how it will be, how it's gonna work out in the end…but I think discussing it definitely helps. Hearing everyone's perspective from different services also helps. It helps you appreciate what others are going through and how this will change practice in our department.

(Participant B6)

Pharmacists also debated how patient outcomes will be affected by cpKPIs, and whether patients' views of pharmacists will be changed.

### Ideas for Overcoming Resistance

Pharmacists provided insight into how resistance to the implementation of cpKPIs could be overcome. Pharmacists suggested that better technology (e.g. tracking clinical activities using a mobile device), simplified documentation, further improvements to the current workload measurement tool, and intermittent or staggered documentation of workload, rather than continuous documentation, would facilitate the documentation of cpKPIs. Pharmacists felt that increased pharmacist coverage and pharmacist hours, more resources, and more technical support (e.g. expansion of unit-based pharmacy technicians) would ease the implementation of cpKPIs. Continuation of current efforts to facilitate the implementation process were also noted, such as ongoing discussions within clinical groups, and education of other health care professionals to promote buy-in. Pharmacists were also interested in obtaining a list of priorities from management, finding ways to show pharmacist impact, and receiving more information regarding plans for the reporting of data captured using Emerald.

## Discussion

Focus groups to explore pharmacists' perceptions of the barriers and facilitators to the implementation of cpKPIs revealed three major themes. The first theme, *resisting the change*, consisted of four categories: documentation challenges, increased workload, practice environment constraints, and competing priorities. The second theme, *embracing cpKPIs* was composed of three categories: seeing the benefit, demonstrating value, and existing supports. Two additional categories, quality versus quantity battle and insights into the future, made up the third theme, *navigating the unknown*.

### Implementation versus Continuation

One concept arising from the data that warrants discussion is the unexpected finding that some pharmacists were confused by the word "implementation". This confusion could be due in part to the fact that each focus group contained pharmacists who had formally begun the implementation process as well as those who had not yet begun the implementation process. At the time of this research, seven out of 15 clinical pharmacy services had implemented cpKPIs. Thus, some pharmacists would not be as familiar as others with how cpKPIs were implemented in this context. However, many pharmacists voiced that they already perform cpKPIs as part of their practice and felt that they are continuing to perform these activities. Pharmacists did not feel that they were implementing new clinical activities into their practice; rather, they felt that they were implementing the process of documenting and monitoring these activities. This finding may explain why pharmacists did not identify lack of knowledge or training as barriers to cpKPI implementation, when these barriers are commonly cited in other studies of pharmacy practice change [27, 28].

### Pharmacists Support cpKPIs, but are Challenged by Documentation and other Changes Associated with the Implementation of cpKPIs

Given that pharmacists mostly felt they already perform cpKPIs, the major perceived change associated with the implementation of cpKPIs was the requirement to document these clinical activities using Emerald. Overall, pharmacists were resistant to this change and thoroughly explored reasons why documentation is a barrier. Several barriers to cpKPI implementation identified by pharmacists in this study have also been reported as barriers to other types of pharmacy practice change. For example, in a systematic review of the barriers and facilitators to the implementation of electronic prescription, pharmacists identified barriers related to the themes of structure (e.g. limitations related to software), process (e.g. interoperability), and outcome (e.g. data accuracy) [29]. These findings are closely related to the barriers associated with the process of documentation that were identified in our study. Additional examples of barriers that have been reported both in our study and in other studies of pharmacy practice change include barriers related to time, staff and resources. For example, pharmacists identified "lack of time" as a leading barrier to pharmacist-led immunization, a "cumbersome and time-consuming process" as a barrier to billing for professional fees by community pharmacists, "inadequate resources" and "inadequate funding" as barriers to pharmacist prescribing in Northern Ireland, and "lack of time" and "lack of staff" as barriers to implementation of pharmaceutical care practice by pharmacists in Kuwait [28, 30–32].

In spite of extensive discussion of the challenges associated with documentation, as well as the barriers of increased workload, practice environment constraints, and competing priorities, 22/26 participants believed that pharmacists at NSHA support the implementation of cpKPIs to either a moderate or great extent. We favour the idea that this impressive pharmacist support for the implementation of cpKPIs will facilitate the cpKPI initiative given that pharmacist attitudes, perceptions and beliefs have been identified as facilitators to pharmacy practice change in other settings [27, 29–32].

### Pharmacists Care About Their Patients and Reflect on the Future of Pharmacy Practice

Analysis of focus group discussions uncovered plenty of evidence that pharmacists genuinely care about their patients and the profession of pharmacy. For example, pharmacists explicitly stated that they want to be able to impact more patients. In multiple instances, pharmacists seemed torn by the need to choose between different options. This included the dilemma of how to prioritize patients, how to best spend their time, choosing between competing priorities, and choosing between providing direct patient care and taking the time to document their clinical activities. Furthermore, pharmacists came up with a broad array of feasible suggestions that would facilitate the implementation of cpKPIs (see Findings; Ideas for Overcoming Resistance). Even though pharmacists were not specifically asked about their insights into the future of pharmacy practice, important insights were revealed in each focus group. This signifies that participants were thinking critically about cpKPIs and deeply considering the future of pharmacy practice.

### Strengths and Limitations

There are significant strengths of this research. Pharmacists at NSHA were well-represented in this study with approximately half of all clinical pharmacists participating in a focus group. Although there are no standard guidelines to determine an appropriate sample size, we anticipated that three to five focus groups made up of five to ten pharmacists each would achieve theoretical saturation and/or data saturation [16, 33]. The fact that only two additional codes were identified after coding the third and final focus group (Table 1) favours the idea that data saturation was indeed achieved. However, it is possible that additional concepts would arise if a fourth or fifth focus group were held; therefore, it is most appropriate to state that theoretical data saturation was achieved.

Pharmacists were both interested in the topic and engaged in discussion, which led to the attainment of rich research data that would not have been captured using quantitative methods. Steps to ensure confidentiality and anonymity, the exclusion of authority figures, the semi-structured nature of questioning, and the opportunity for participants to add final thoughts at the end of each focus group promoted the ability of participants to contribute freely and openly during focus group discussions. In most cases, the moderator had an established relationship with research participants. It is possible that participants were either more or less likely to contribute to focus group discussion due to this established relationship. It is also possible that this established relationship may have facilitated or hindered recruitment of participants. Additional steps were taken to ensure credibility, reliability, transferability, and trustworthiness of this research (see Methods).

Although a large number of pharmacists participated in this study, some sites were better represented than others. Due to the voluntary nature of participation in focus groups, it is possible that pharmacists who chose not to participate have different views than those who did volunteer to participate. However, given the wide distribution of thoughts and opinions that were discussed by participating pharmacists, we believe that this group is representative overall. Furthermore, most pharmacists responded with specific reasons (e.g. scheduling difficulties) to explain why they could not attend a focus group.

### Relevance to Practice and Next Steps

We uncovered previously unidentified barriers and facilitators to the cpKPI implementation process at NSHA. This information will be used to ease the implementation process locally, thereby increasing its success with the overall goal of allowing more patients to be impacted by pharmacists, and the quality of care to be enhanced. Our findings will also be used to facilitate the training of new pharmacy staff and may be used to direct the potential province-wide adoption of cpKPIs by other hospitals in the new NSHA.

By assessing the barriers and facilitators to the implementation of cpKPIs, our findings serve as an important step in the knowledge-to-action process and will be used to select, tailor, and implement interventions to facilitate the application of knowledge into practice [34]. Given the complexity of behaviour change, comprehensive approaches that target various levels of health care are required [35, 36]. Knowledge obtained from the focus groups has been, and will continue to be, applied in a variety of ways to improve the implementation of cpKPIs at NSHA. To begin this complex process, the cpKPI staff pharmacist and the cpKPI Leadership Team analyzed barriers related to the theme *resisting the change* to generate ideas for how to overcome specific barriers. For example, focus group participants identified that challenges associated with the documentation of cpKPIs included unclear definitions of cpKPIs and uncertainty regarding the validity of data entered into Emerald. Based on this knowledge, the cpKPI staff pharmacist initiated a bi-weekly email to front-line pharmacists that contains an anonymous questionnaire asking pharmacists to indicate how they would record activities in Emerald based on a specific clinical scenario. Follow-up emails with pooled questionnaire results were then distributed to staff to provide a consensus approach to the documentation of clinical activities and to clarify the definitions of cpKPIs. The goal of this intervention was to reduce confusion around documentation of clinical activities, create greater consistency of documentation amongst staff and ultimately improve the validity of the data. Other examples of interventions designed to overcome barriers related to documentation of cpKPIs include modifications to Emerald and the generation of tools that can be used to help track and record which cpKPIs have been completed for each patient. Interventions have also been trialed to address barriers related to *competing priorities* as identified by focus group participants. Such interventions include defining an approach to the prioritization of activities that is consistent and agreed upon by all members, and focused discussions to improve the handover of patient information amongst team members. To continue to improve the implementation of cpKPIs, additional interventions will be designed based on knowledge obtained from the focus groups. To further inform the choice of behaviour change strategy, there are a number of frameworks in which our findings could be considered. For example, our findings could be interpreted in the context of the behaviour change wheel and the theoretical domains framework for behaviour change to improve cpKPI implementation efforts at any given organization [37, 38].

As most Canadian health authorities are either in the early stages of cpKPI implementation or have not yet begun to formally implement cpKPIs, it is our hope that this information will also be used by other organizations to inform this process on a national and/or international level. More generally, this research will increase awareness of the cpKPI initiative and the evidence-based activities performed by clinical pharmacists, which will further promote the profession of pharmacy. In addition, since the barriers and facilitators identified by this research will be used to design interventions to facilitate the uptake of and improve the ability of pharmacists to incorporate more cpKPIs into their practice, this will promote pharmacist clinical activities and positively impact patient care.

## Supporting Information

S1 Appendix Focus Group Topic Guide. (DOCX)

S2 Appendix Thematic analysis: themes, categories, and codes. (DOCX)
