# Supplementary material for: Pharmacists' Perceptions of the Barriers and Facilitators to the Implementation of Clinical Pharmacy Key Performance Indicators
Source: PLoS One. 2016 Apr 4;11(4):e0152903. doi: 10.1371/journal.pone.0152903 (PMC4820256; doi:10.1371/journal.pone.0152903)
Supplement: S1 Appendix — (DOCX) [file pone.0152903.s001.docx]

**S1 Appendix. Focus Group Topic Guide.**

Question 1

Can I start by asking you what your thoughts are about cpKPI implementation at CDHA?

Question 2

What would help or facilitate(has helped or facilitated) your ability to implement cpKPIs?

Prompts

-Are there any resources or tools you can think of?

-Is there anything that has been or could be changed to make this easier?

Question 3

What would get(has gotten) in the way of, or be(been) a barrier to, your ability to implement cpKPIs?

Prompts

-What is the biggest thing standing in your way? Your biggest frustration/problem/worry?

-Is there some aspect you find most difficult?

Question 4

Please circle the most appropriate response to the statement below:

"Pharmacists at Capital Health support the implementation of cpKPIs"

to a great extent to a moderate extent to a slight extent not at all

Prompts

-Do you feel there is a culture of support among pharmacists at CDHA?

-Do you get the feeling CDHA pharmacists think this initiative is important or unimportant? Or neither?

Question 5

How do you think cpKPI implementation may affect patients at CDHA?

Prompts

-Do you think patients will benefit or be harmed by cpKPI implementation? Neither?

-Do you think patient outcomes may be affected? how so?

-Do you think the implementation of cpKPIs could influence the way that patients view pharmacists at CDHA?

Question 6

What do you think the most important elements of this discussion have been?

Question 7

Is there anything else we missed talking about?
