# Supplementary material for: Pharmacists' Perceptions of the Barriers and Facilitators to the Implementation of Clinical Pharmacy Key Performance Indicators
Source: PLoS One. 2016 Apr 4;11(4):e0152903. doi: 10.1371/journal.pone.0152903 (PMC4820256; doi:10.1371/journal.pone.0152903)
Supplement: S2 Appendix — (DOCX) [file pone.0152903.s002.docx]

**S2 Appendix. Thematic analysis: themes, categories, and codes.**

Table 1. *Resisting the change*: categories and codes.

| **Theme** | ***Resisting the change*** | | | |
| --- | --- | --- | --- | --- |
| **Category** | **Documentation challenges** | **Increased workload** | **Practice environment constraints** | **Competing priorities** |
| **Codes** | Aggravating | Case costing (takes time) | Challenges unique to clinical area | 2 North |
|  | Case costing (process of entering) | Difficult to do ALL the cpKPIs | Chaotic work environment | Antimicrobial stewardship pharmacist |
|  | Definitions of cpKPIs | Frustrating (so much to do) | Coverage | Distribution |
|  | Difficult to keep up with Emerald | Overloaded with things to do | Different sites are different | Emerald versus cpKPIs |
|  | Documentation is confusing | Quality of Emerald (time to do good job) | Electronic discharge medication reconciliation tool | Emerald versus other activities |
|  | Fear of job loss | Time | Finding things | Expectations |
|  | Forget to document | Time to document in Emerald | Hours worked | Expectations of other health care professionals |
|  | Forget to track | Too much other change | Money | cpKPIs already outdated (new things we should do?) |
|  | Frustrating (to have to do it) |  | No best possible medication history | cpKPIs versus other activities |
|  | Guilt |  | No computer | Learners |
|  | Number of patients to document |  | Not enough communication | Medication calendars |
|  | Problems selecting patient list |  | Not enough staff | Not going to change practice now |
|  | Process of documentation |  | Not enough support in place | Order entry |
|  | Requirement for continuous documentation |  | Not every patient needs every cpKPI | Priorities of management |
|  | Resentment |  | Number of patients | Research |
|  | Timing of documentation |  | Others not using Emerald | Rounds |
|  | Tired |  | Patient location | Teaching |
|  | Too much clicking/scrolling |  | Problems created by joint competencies | Writing orders |
|  | Tracking clinical activities |  | Resources |  |
|  | Unsupportive opinions of Emerald |  | Timing of discharge medication reconciliation |  |
|  | Validity of Emerald (everyone doing something different) |  | Timing of pharmacist-patient interaction |  |
|  |  |  | Top three cpKPIs aren't service-specific |  |

Table 2. *Embracing clinical pharmacy key performance indicators*: categories and codes.

| **Theme** | ***Embracing clinical pharmacy key performance indicators*** | | |
| --- | --- | --- | --- |
| **Category** | **Existing supports** | **Seeing the benefit** | **Demonstrating value** |
| **Codes** | Accountability | Align expectations | Confusion over role |
|  | Communication with other health care professionals | Barriers can be worked with | Data may help justify positions |
|  | Dedicated cpKPI staff pharmacist | Changed opinions | Documentation is important |
|  | Descriptions within Emerald | Culture of support for cpKPIs | Having the data may lead to change |
|  | Discussions | Current practice is inconsistent | Increase patient awareness of pharmacist role |
|  | Documentation serves as a reminder | Exciting | cpKPIs might help others know what we do |
|  | Emerald reminder | Focus clinical activities | Prove ourselves |
|  | Emerald training sessions | Hopeful | Value of pharmacist |
|  | Feedback | Identify activities that can be shifted to others |  |
|  | Handover | cpKPIs are evidence-based |  |
|  | Improvements to Emerald | cpKPIs are important |  |
|  | Joint competencies can support pharmacists | cpKPIs are what pharmacists do and have done |  |
|  | Knew about cpKPIs early | cpKPIs make sense |  |
|  | Learning from peers | cpKPIs may benefit patient care |  |
|  | Promotion | cpKPIs may make practice more consistent |  |
|  | Standardization of time per task | cpKPIs re-focus/remind |  |
|  | Support of organization | Personal support for cpKPIs |  |
|  | Technical support | Value in implementation |  |
|  | Tools for tracking |  |  |

Table 3. *Navigating the unknown*: categories and codes.

| **Theme** | ***Navigating the unknown*** | |
| --- | --- | --- |
| **Category** | **Quality versus quantity battle** | **Insights into the future** |
| **Codes** | Complexity | Emerald and jobs |
|  | More care for fewer patients versus less care for more patients | Evaluation |
|  | Not all cpKPIs are equal | Hopeful |
|  | Not every patient needs every cpKPI | Individual right to choose versus streamline |
|  | Number of patients | Limit practice |
|  | Prioritize patients | New health authority |
|  | Quality of cpKPIs | Patient outcomes |
|  | Want to see more patients | Patient views of pharmacists |
|  | Where to spend time | Practice change |
|  |  | Quality of cpKPIs |
|  |  | Reporting of Emerald data |
|  |  | Target |
|  |  | Which cpKPI? |
|  |  | Who's responsibility is it? |
|  |  | Validity of Emerald (what will it mean?) |
